# Artificial intelligence applications for pre-implantation kidney biopsy pathology practice: a systematic review

**DOI:** 10.1007/s40620-022-01327-8

**Published:** 2022-04-19

**Authors:** Ilaria Girolami, Liron Pantanowitz, Stefano Marletta, Meyke Hermsen, Jeroen van der Laak, Enrico Munari, Lucrezia Furian, Fabio Vistoli, Gianluigi Zaza, Massimo Cardillo, Loreto Gesualdo, Giovanni Gambaro, Albino Eccher

**Affiliations:** 1 Division of Pathology, Central Hospital Bolzano, Bolzano, Italy; 2 Department of Pathology and Clinical Labs, University of Michigan, Ann Arbor, MI USA; 3 Department of Diagnostics and Public Health, University and Hospital Trust of Verona, Verona, Italy; 4 Department of Pathology, Radboud University Medical Center, Nijmegen, The Netherlands; 5 Pathology Unit, Department of Molecular and Translational Medicine, Spedali Civili-University of Brescia, Brescia, Italy; 6 Department of Surgical, Oncological and Gastroenterological Sciences, Unit of Kidney and Pancreas Transplantation, University of Padua, Padua, Italy; 7 Division of General and Transplant Surgery, University of Pisa, Pisa, Italy; 8 Department of Nephro-Urology, Nephrology, Dialysis and Transplant Unit, University of Foggia, Foggia, Italy; 9 National Transplant Center, Rome, Italy; 10 Nephrology, Dialysis, and Transplantation Unit, Department of Emergency and Organ Transplantation, University of Bari Aldo Moro, Bari, Italy; 11 Department of General Medicine, Renal Unit, University and Hospital Trust of Verona, Verona, Italy; 12 Department of Pathology and Diagnostics, University and Hospital Trust of Verona, P.le Stefani n. 1, 37126 Verona, Italy

**Keywords:** Digital pathology, Pre-implantation biopsy, Kidney biopsy, Artificial intelligence, Review, Transplantation

## Abstract

**Background:**

Transplant nephropathology is a highly specialized field of pathology comprising both the evaluation of organ donor biopsy for organ allocation and post-transplant graft biopsy for assessment of rejection or graft damage. The introduction of digital pathology with whole-slide imaging (WSI) in clinical research, trials and practice has catalyzed the application of artificial intelligence (AI) for histopathology, with development of novel machine-learning models for tissue interrogation and discovery. We aimed to review the literature for studies specifically applying AI algorithms to WSI-digitized pre-implantation kidney biopsy.

**Methods:**

A systematic search was carried out in the electronic databases PubMed-MEDLINE and Embase until 25th September, 2021 with a combination of the key terms “kidney”, “biopsy”, “transplantation” and “artificial intelligence” and their aliases. Studies dealing with the application of AI algorithms coupled with WSI in pre-implantation kidney biopsies were included. The main theme addressed was detection and quantification of tissue components. Extracted data were: author, year and country of the study, type of biopsy features investigated, number of cases, type of algorithm deployed, main results of the study in terms of diagnostic outcome, and the main limitations of the study.

**Results:**

Of 5761 retrieved articles, 7 met our inclusion criteria. All studies focused largely on AI-based detection and classification of glomerular structures and to a lesser extent on tubular and vascular structures. Performance of AI algorithms was excellent and promising.

**Conclusion:**

All studies highlighted the importance of expert pathologist annotation to reliably train models and the need to acknowledge clinical nuances of the pre-implantation setting. Close cooperation between computer scientists and practicing as well as expert renal pathologists is needed, helping to refine the performance of AI-based models for routine pre-implantation kidney biopsy clinical practice.

**Supplementary Information:**

The online version contains supplementary material available at 10.1007/s40620-022-01327-8.

## Introduction

Transplant nephropathology is a highly specialized field of pathology comprising both the evaluation of organ donor biopsy for organ allocation and post-transplant graft biopsy for assessment of rejection or graft damage. Recognizing and quantifying various organ structures and subtle histopathological features, and correlating these findings with clinical parameters are required to be of use for donor or recipient management in the current era of precision medicine [1]. Added challenges in this field include the increasing demand to render complex diagnoses in kidney biopsy samples, pressure of time constraints in the case of pre-implantation biopsy for organ allocation, and the lack of specifically trained nephropathologists. Moreover, when dealing with kidney transplant pathology, a distinction should be made between pre-transplant or time-zero donor biopsy and protocol biopsy on a graft kidney as the clinical setting and related challenges of these two scenarios are quite different. In a graft biopsy, simultaneous assessment of rejection and/or chronic organ damage is made by means of a multiplicity of ancillary techniques (e.g., immunohistochemistry for C4d, special stains for fibrosis or deposits, immunofluorescence or molecular investigations) on a biopsy specimen collected in optimal conditions with an expected turn-around-time (TAT) for results of days to weeks, usually assessed by reference pathologists with expertise in the field. On the other hand, pre-implantation biopsy for organ suitability to transplantation is usually assessed via frozen section (FS) or microwave rapid processing protocols of slides with no aid of ancillary techniques or only very few, with a time-critical diagnostic TAT diagnosis rendered by a general pathologist with relatively little specific expertise in this field. The Banff group developed definitions for features to be evaluated in kidney biopsy and scoring systems, but reproducibility even among experts remains an issue [2], and studies on correlation of pre-implantation biopsy and graft outcome have highlighted that expertise is crucial in predicting the true state of an organ and subsequent outcome [3, 4].

The introduction of digital pathology with whole-slide imaging (WSI) in clinical research, trials and practice has catalyzed the application of artificial intelligence (AI) in histopathology, with the development of novel machine-learning models for tissue interrogation and discovery. Such advances in technology offer the potential to improve our ability to classify disease, quantify morphological alterations more accurately, discover correlations with pathogenesis and clinical data, and predict disease outcome with new prediction models [5]. As reported in a recent review by Farris et al., interest in the application of AI algorithms towards the topic of kidney transplant has grown, as demonstrated by the increasing number of publications retrieved from PubMed with the keywords “computer & pathology & image & analysis” [6]. As highlighted from previous work [7], most efforts have been devoted to the post-transplant graft biopsy with algorithms aiming to quantify and describe features of rejection such as immune infiltrate, or to quantify chronic organ damage in terms of interstitial fibrosis with special stains. By comparison, studies regarding the application of AI algorithms to the pre-implantation biopsy are scarce and more focused on fewer simpler tasks, such as counting glomeruli, sclerotic glomeruli, vascular structures, and quantification of interstitial fibrosis. Nevertheless, digital pathology with WSI has already been deployed in the pre-implantation setting for consultation and training [8–11].

In this work we systematically reviewed the literature searching for all studies applying AI algorithms to digitized slides of pre-implantation kidney biopsy. The various AI-based algorithms developed and deployed for this purpose are discussed, as are the potential benefits and main challenges encountered.

## Methods

### Search strategy

A systematic search was carried out in the electronic databases PubMed-MEDLINE and Embase until 25th September, 2021 with a combination of the key terms “kidney”, “biopsy”, “transplantation” and “artificial intelligence” and their aliases (see complete search strategy in the Supplementary Table 1). No language filters were applied. Two authors screened all retrieved items after removal of duplicates with the aid of the web-app Rayyan QRCI [12]. Briefly, the two authors screened abstracts blinded to each other’s decision and after finishing they compared the results of the screening. Disagreement was resolved by consensus. Then authors read the full text and decided on final inclusion, with consultation of a third author in case of disagreement.

### PICOS analysis

Pre-implantation kidney biopsy (P) employing AI algorithms coupled with WSI (I) were compared to conventional assessment (C) to determine potential concordance rates and performance of algorithms (O). Studies concerning protocol graft biopsy for rejection, not dealing with AI algorithms or not deploying WSI, together with studies represented only by abstracts were excluded. Disagreement on article screening and final inclusion was resolved with the participation of a third reviewer. References listed in the excluded but relevant articles, as well as recent reviews on the topic were checked for additional studies that might be potentially included. Full-texts of relevant studies were acquired and checked, and data from included studies were extracted and summarized. Extracted data were: author, year and country of the study, type of biopsy features investigated, number of cases, type of algorithm deployed, main results of the study in terms of diagnostic outcome, and the main limitations of the study.

## Results

After removal of duplicates, a total of 5761 publications were screened. Of these, 184 were selected as potentially relevant, and only seven were finally included [13–19]. Reasons for exclusion included: 23 with only an abstract available, 115 not using WSI, 11 not specifically concerned with the transplant setting, and 28 not dealing with pre-implantation biopsy. Only a few studies we identified regarding AI algorithms coupled with WSI were dedicated to pre-implantation biopsies, and they mainly focused on the detection and classification of glomerular structures and to a lesser extent tubular and vascular structures. Included studies reported the performance of the algorithms in terms of sensitivity and specificity or correlation with pathologists’ assessment as outcome measures. The included studies are summarized in Table 1.

**Table 1:** Summary of included studies

References	Histological feature	*N* and type of cases	Type of algorithm	Main results
Altini et al. [15] (Italy)	Glomeruli detection and classification	26, PAS	CNN architecture for semantic segmentation with two models	Global accuracy higher than 0.98; precision in classifying healthy and sclerosed glomeruli ranging 0.834–0.935 and 0.806–0.976
Bevilacqua et al. [16] (Italy)	Tubuli and vessels detection	10, PAS	Two-layer, error back-propagation ANN	Accuracy higher than 0.93, precision higher than 0.88 in validation set and higher than 0.91 in test set
Cascarano et al. [17] (Italy)	Glomeruli detection and classification	26, PAS	Shallow ANN	0.99 accuracy, 1.00 precision
Marsh et al. [13] (USA)	Glomeruli detection and classification	47, FS H&E	Patch-based and fully CNN model	Fully CNN model with greater correlation with percent global glomerulosclerosis (*R*^2^ = 0.828); robust to preparation artifacts
Marsh et al. [14] (USA)	Glomeruli detection and classification	98 FS and 51 permanent sections, H&E	Deep-learning model based on the model of previous study	Higher correlation of model with ground truth annotations (*r* = 0.916) than on-call pathologists; lower quota of kidneys classified as to be discarded respect to on-call pathologists
Salvi et al. [18] (Italy)	Vessels and interstitial fibrosis detection	65, PAS and trichromic	RENFAST model: semantic segmentation CNN model	Accuracy 0.89–0.94 for vessel detection with Dice score 0.83–0.84; accuracy 0.92 for interstitial fibrosis; 2 min of computation time against 20 min for pathologists
Salvi et al. [19] (Italy)	Glomeruli and tubuli detection and classification	83, PAS	RENTAG model: semantic segmentation CNN model	Dice score of 0.95 and 0.91 for glomeruli and tubuli detection; 100% sensitivity and PPV; little time of computation required

*ANN* artificial neural network, *CNN* convolutional neural network, *FS* frozen section, *H-AI-L* human AI loop, *H&E* hematoxylin & eosin, *NS* not stated, *PAS* Periodic-acid Schiff, *PPV* positive predictive value

### Assessment and concordance rates

Marsh et al. in 2018 trained a deep-learning model based on a convolutional neural network (CNN) utilizing 48 WSI cases of kidney biopsy processed by FS. These authors tested the performance of their model to detect and classify normal and sclerosed glomeruli, demonstrating performance comparable to that of expert pathologists and reported robustness of their algorithm against slide preparation artifacts. In the training phase, normal and sclerosed glomeruli were annotated in the test set of biopsies obtained from 20 kidneys of 17 donors, with a range of glomerulosclerosis 1–72%. Annotation was undertaken by a senior resident and amended by a specialist nephropathologist, thus underscoring the importance of expert annotation in training AI algorithms. Two different CNN models were tested, an image patch-based and full WSI convolutional model. The fully convolutional model showed the best performance by more accurately measuring the proportion of correctly classified sclerosed glomeruli. The fully convolutional model also showed greater correlation with the percent global glomerulosclerosis evaluated (*R*^2^ = 0.828) compared with the patch-based model (*R*^2^ =  − 0.491)[13]. Moreover, as these authors stressed, the model was not only robust to slide preparation artifacts such as tissue folds, but it was also capable of running the analysis within a time-span satisfactory for a FS request. Additional data reported by the same group of investigators in 2021 [14], where a deep-learning model was based on the model of their previous study, were challenged on a larger population of mixed wedge and core kidney biopsy cases (98 FS and 51 permanent sections). Again, the training set of biopsies was annotated by three certified pathologists with expertise in renal transplant pathology. Glomeruli counts were compared against annotation ground truth, with accuracy assessed by Pearson correlation coefficient r and root-mean-square error (RMSE). Corresponding quantities for percent global glomerulosclerosis were computed for on-call pathologists’ estimates, and those values were compared with the model’s performance [14]. Moreover, the authors used Cohen’s Kappa to test the concordance of the model with the ground truth derived from pathologists at the cut-off of 20% glomerulosclerosis for organ discard. The model correlated very well with the pathologists’ annotations, with a correlation coefficient higher than 0.900. Interestingly, when the pathologists were asked to check and correct the classification of glomeruli after the model had been run on a subset of 25 cases with different grades of glomerulosclerosis by visualizing the histology images with overlaid model-generated glomeruli classifications, the correlation with ground truth improved with respect to both the original on-call pathologists’ reports and to the model alone. This was underscored by the authors as indirect proof that their model could potentially be incorporated into routine clinical practice [14].

A group of Italian researchers shared slightly different semantic segmentation CNN models that were trained on small datasets of pre-implantation kidney biopsies stained with Periodic acid-Schiff (PAS) to detect vessel and tubular structures, starting with a lumen as an object and then extracting nuclei and membranes to classify the structure [16], or detect healthy and sclerosed glomeruli [15, 17]. Early work of this group utilized a binary classifier (vessel vs tubule) classification that was performed with Back Propagation Neural Network (BPNN) and Haralick texture features [16]. The classification provided by the algorithm in terms of counting vascular and tubular structures was compared to manual counting provided by an expert nephropathologist. They tested four different approaches to reduce false positives detected by the algorithm. The model always detected more vessels and tubules than the human expert, with a final precision in the test set of 0.91. These authors concluded that such an algorithm could be of aid to a pathologist assessing these structures, given that their task would be simplified and limited to a final check on the output of the algorithm [16]. Subsequent research by this Italian group focused on glomerular structure detection and classification as sclerosed or normal. The detection of glomeruli and their classification was based on the evaluation of several features, such as the sum of the area related to Bowman’s capsule, blood vessel area and the inter-capillary space, diameter, and texture features, with the use of two well-known texture analysis algorithms; namely, Local Binary Pattern (LBP) and Haralick features. A total of 150 features were extracted and reduced to 95 by a principal component analysis, and a shallow CNN final model was applied. The best performance achieved an accuracy and precision of 0.98, and the authors reported that the cases of misclassified glomeruli were reviewed by an expert pathologist who acknowledged the challenge of classifying such images and indicated that in routine practice such image fields would not be considered for evaluation [17]. In their subsequent work, the authors replaced the last layer of both SegNet [20] and DeepLab v3 +  [21] networks with a pixel-wise classification layer with three output classes (background, sclerotic glomeruli and non-sclerotic glomeruli) to accomplish the same task. Both models worked better in the detection of non-sclerosed glomeruli and background tissue, i.e., the SegNet-based model yielded a better F-score for both classes of glomeruli , while the DeepLab v3 + -based model had a better F-score for non-sclerotic glomeruli and a slightly worse F-score for sclerotic glomeruli [15]. In both studies the authors stressed the importance of expert human annotation for reliably training models and the potential application of such computer-aided diagnosis tools for practicing pathologists. They also acknowledged that non-expert pathologists tend to overscore glomerulosclerosis, which may lead to excessive discarding of kidneys [3, 4].

### Algorithms

Salvi et al. also designed algorithms to classify glomeruli, quantify tubular atrophy, detect blood vessels and quantify interstitial fibrosis on PAS-stained slides [18, 19]. Their algorithm, called RENFAST (Rapid Evaluation of Fibrosis And vessels Thickness), deals with semantic segmentation using CNN and employs U-Net architecture with a ResNet34 backbone [22]. A dataset of 65 kidney biopsies stained with PAS and a trichrome stain for fibrosis were manually annotated by an expert renal pathologist. The RENFAST algorithm yielded a balanced accuracy of 0.89 and a precision of 0.92 utilizing the test set for blood vessel detection and of 0.92 and 0.91, respectively, for fibrosis quantification; of note, average absolute errors between manual and computational assessment were lower than 2.5%. Moreover, these authors reported that their computational time was around 2 min, significantly less than the 20 min required by the pathologist to manually evaluate arterial and interstitial fibrosis [18]. The algorithm, named RENTAG (Robust Evaluation of Tubular Atrophy & Glomerulosclerosis), deployed by another study group [19], consisted of three modules: PAS normalization, glomerulosclerosis assessment, and tubular atrophy quantification. This algorithm was based on a deep convolutional network, with pixels labeled into three different classes depending on whether they belonged to healthy glomeruli/tubuli, sclerotic glomeruli/atrophic tubuli or other components of renal tissue and then subjected to a post-processing procedure. A total of 83 needle-core biopsy cases stained with PAS were used and annotated by two trained pathologists. The algorithm achieved high sensitivity and positive predictive value concerning correct classification of normal and sclerosed glomeruli and normal and atrophic tubuli, with a reported total time needed to run the analysis of around 3 min.

## Discussion

As expected, we retrieved very few papers concerning AI and WSI slides of pre-implantation biopsy, as anticipated in previous reviews [7]. This is not surprising, considering that in many settings pre-implantation biopsies are evaluated with rapid protocols that are more prone to artifacts and pose challenges in standardization. Moreover, in the pre-implantation setting, the features of interest to be assessed are almost exclusively based upon morphology, given that any sort of score (Karpinski-Remuzzi [23, 24], donor score [4], Banff [2]) is based on relatively simple tasks such as counting and classifying glomeruli and quantifying interstitial fibrosis, tubular atrophy and vascular narrowing, usually with no availability of ancillary techniques such as special stains and immunohistochemistry. On the other hand, the development and testing of AI algorithms offers an opportunity for standardization. Indeed, many of the studies encountered in this review were about “narrow AI” because they dealt only with these elementary tasks (e.g. enumerating and classifying glomeruli and quantifying fibrosis). A brief overview of these studies is provided in Supplementary Table 2.

Animal model-derived samples were deployed in several studies, most of which dealt with glomerular detection [25–33], or unspecified human kidney biopsies with no reference to the pre-implantation setting or specific pathology [34–38]. Only three larger-sized studies applied segmentation CNN models to the detection and simultaneous classification of multiple renal structures, not only glomeruli but also different kinds of tubuli and vessels [39–41]. In particular, Hermsen et al. [39] developed a multiclass segmentation CNN that achieved a high Dice coefficient for all of the segmentation classes (“glomeruli,” “sclerotic glomeruli,” “empty Bowman’s capsules,” “proximal tubuli,” “distal tubuli,” “atrophic tubuli,” “undefined tubuli,” “capsule,” “arteries,” and “interstitium”), with the best results being yielded by glomerular detection, followed by tubules as a whole class and then the interstitium. Their AI-based algorithm was validated not only in a single-center experimentation setting, but also on biopsy material from another center and on nephrectomy specimens. Moreover, the study by Hermsen and colleagues is the only one to correlate results of the algorithm to expert pathologists’ assessment according to Banff categories for tubular damage and interstitial fibrosis. In another study by Jayapandian et al., albeit unrelated to the transplantation setting, the multicenter Nephrotic Syndrome Study Network (NEPTUNE) dataset of digitized renal biopsies was evaluated for the feasibility of deep learning approaches to automatically segment utilizing four stains [40]. Five nephropathologists were employed for annotations and 29 centers were enrolled to provide material whereby 20 deep-learning models were tested, making this work one of the most powered to date concerning kidney histology and AI. PAS proved to be the most suitable stain for computer-aided diagnosis and produced the highest concordance with expert evaluation for all the investigated structures, while silver-based stains yielded the worst results. Bouteldja et al. tested a CNN multiclass segmentation model on a large series of tissue specimens, derived from a plethora of animal models of various kidney diseases and a small subset of human tissue. All their preparations were PAS stained and six classes were tested: tubule, full glomerulus, glomerular tuft, artery (including intima and media, but excluding adventitia), arterial lumen, and vein. The best results in terms of average precision in detection was achieved for glomeruli, while accuracy for vascular structures was the lowest; moreover, performance on human tissue was intermediate. The authors also underscored the importance of training pathologists to ensure high quality annotations, and highlighted that good algorithm performance can be achieved with database heterogeneity, such as utilizing a variety of animal species and renal diseases [41].

It is noteworthy that all the included studies stressed the impact of their experimental setting: i.e., pre-implantation kidney biopsy stained only with PAS or H&E in the case of frozen sections, aiming to adhere as much as possible to the real-life practice of pathologists, with a subset aiming to use AI-aided evaluation of the Karpinski score [15–17]. Only one study explicitly compared the performance of the algorithm virtually, in terms of decision to discard a kidney with the cutoff of 20% glomerulosclerosis with the results of the on-call pathologists and the annotations made by expert pathologists [14], thus directly translating the deployment of the algorithm in clinical practice. However, all the other included studies recapitulate and discuss briefly the importance of an accurate and reliable evaluation of the single features of the kidney biopsy, its difficulties, the need for specific expertise and the relevant consequences in terms of erroneous discard. Nevertheless, despite the fact that deep-learning models are capable of excellent detection and classification of renal structures, for such an algorithm to be approved for clinical use it must first be tested and validated prior to clinical deployment. AI could become extremely helpful for practicing pathologists, especially given the projected decrease in the pathology workforce and continued demand for specialization [42]. All included studies also highlighted the need to adjust AI models to work with WSI, in anticipation of more pathology laboratories ultimately transitioning to a fully digital workflow.

## Conclusion

Within the last decade many studies have been published testing AI algorithms developed for the detection and classification of elementary structures in kidney biopsy material. However, very few of these studies specifically designed AI-based models to optimize the evaluation of renal biopsies in the pre-implantation setting. It is foreseeable that in the near future more multicenter projects will provide additional contributions to the transplantation field. Closer cooperation between computer scientists and practicing as well as expert renal pathologists is needed, helping to refine the performance of AI-based models for routine pre-implantation kidney biopsy clinical practice.

## Supplementary Information

Below is the link to the electronic supplementary material.

Supplementary file1 (DOC 30 kb)

Supplementary file2 (DOCX 15 kb)

Supplementary file3 (DOCX 18 kb)

## Data Availability

Not applicable.
