# Supplementary material for: Artificial intelligence applications for pre-implantation kidney biopsy pathology practice: a systematic review
Source: J Nephrol. 2022 Apr 19;35(7):1801–8. doi: 10.1007/s40620-022-01327-8 (PMC9458558; doi:10.1007/s40620-022-01327-8)
Supplement: Supplementary file 1 — Supplementary file1 (DOC 30 kb) [file 40620_2022_1327_MOESM1_ESM.doc]

**Screening**

**Included**

**Eligibility**

**Identification**

Records identified through database searching
(n = 6382)

Records after duplicates removed
(n = 5761)

Records screened
(n = 1787)

Records excluded
(n = 1496)

Full-text articles assessed for eligibility
(n = 184)

Full-text articles excluded, with reasons
(n = 177)

- Not WSI (n = 115)
- Not pre-implant (n= 28)
- Only abstract (n = 23)
- Not transplant (n = 11)

Studies included in qualitative synthesis
(n = 7)
