# Supplementary material for: Artificial intelligence applications for pre-implantation kidney biopsy pathology practice: a systematic review
Source: J Nephrol. 2022 Apr 19;35(7):1801–8. doi: 10.1007/s40620-022-01327-8 (PMC9458558; doi:10.1007/s40620-022-01327-8)
Supplement: Supplementary file 2 — Supplementary file2 (DOCX 15 kb) [file 40620_2022_1327_MOESM2_ESM.docx]

**Supplementary Table 1. Search strategies.**

| **Pubmed** |  |
| --- | --- |
|  | #1 "image"[Title/Abstract] AND "analysis"[Title/Abstract]  #2 "artificial"[Title/Abstract] AND "intelligence"[Title/Abstract]  #3 "morphometry"[Title/Abstract] OR "morphometric"[Title/Abstract] OR "histomorphometric"[Title/Abstract] OR "AI"[Title/Abstract] OR "algorithm*"[Title/Abstract] OR "neural network"[Title/Abstract] OR "neural networks"[Title/Abstract] OR "convolutional"[Title/Abstract] OR "deep-learning"[Title/Abstract] OR "deep-learning"[Title/Abstract] OR "computational"[Title/Abstract] OR "computerized"[Title/Abstract] OR "automated"[Title/Abstract] OR "machine-learning"[Title/Abstract] OR "machine-learning"[Title/Abstract]  #4 #1 OR #2 OR #3  #5 "kidney"[Title/Abstract] OR "renal"[Title/Abstract]  #6 "transplant*"[Title/Abstract] OR "graft*"[Title/Abstract] OR "allograft*"[Title/Abstract]  #7 #4 AND #5 AND #6  #8 "Artificial Intelligence"[Mesh]) AND ("Kidney Transplantation"[Mesh]  #9 #7 OR #8 |
| **Embase** |  |
|  | #1 image AND analysis  #2 artificial AND intelligence  #3 morphometry OR morphometric OR histomorphometric OR AI OR algorithm* OR “neural network” OR “neural networks” OR convolutional OR “deep learning” OR deep-learning OR computational OR computerized OR automated OR “machine learning” OR machine-learning  #4 #1 OR #2 OR #3  #5 kidney OR renal  #6transplant* OR graft* OR allograft*  #7 #4 AND #5 AND #6 |
