# Supplementary material for: Artificial intelligence applications for pre-implantation kidney biopsy pathology practice: a systematic review
Source: J Nephrol. 2022 Apr 19;35(7):1801–8. doi: 10.1007/s40620-022-01327-8 (PMC9458558; doi:10.1007/s40620-022-01327-8)
Supplement: Supplementary file 3 — Supplementary file3 (DOCX 18 kb) [file 40620_2022_1327_MOESM3_ESM.docx]

**Supplementary Table 2. Summary of background studies excluded from review.**

| **Author, year (Country)** | **Histological feature** | **N and type of cases** | **Type of algorithm** | **Main results** |
| --- | --- | --- | --- | --- |
| Bouteldja et al., 2021 (Germany) | Tubuli, glomeruli, arteries, veins, arterial lumens detection | 168, mice, human, others, PAS | CNN model based on U-Net architecture | Accuracy for glomeruli ranging 0.91-1.00, for tubules 0.88-0.95, for veins 0.80-1.00, for arteries 0.73-0.88 and for arterial lumens 0.67-0.81 |
| Bueno et al., 2020 (Spain) | Glomeruli detection and classification | 47, human, PAS | SegNet-VGG19 + AlexNet | 0.98 accuracy for detection and 0.99 for classification |
| Bukowy et al., 2018 (USA) | Glomeruli detection | 87, rat and human, trichromic | Region-based CNN | 0.99 accuracy, precision ranging 0.80-0.96 |
| Gadermayr et al., 2017 (Germany) | Glomeruli detection | 8, mouse, PAS | Weakly supervised patch-based detection and segmentation | 51% correct detection with Dice score >0.80 |
| Gallego et al., 2018 (Spain) | Glomeruli detection | 108, human, PAS | Pre-trained AlexNet model | Precision 0.88 |
| Ginley et al., 2017 (USA) | Glomeruli detection | NS, rat, various stains | Unsupervised texture segmentation  method with Gabor filter banks | Average sensitivity and specificity of 0.88 and 0.96 |
| Ginley et al., 2019 (USA) | Glomeruli detection, nuclear and luminal detection | 79, human and rat, PAS | Iterative convolutional learning interface, human-artificial-intelligence-loop | Balanced accuracy 0.93 (sensitivity 0:88; specificity 0.99) for glomerular detection; Cohen’s Kappa ranging 0.48-0.68 with pathologists on classification of diseased glomeruli |
| Hermsen et al., 2019 (Netherlands) | Tubuli and glomeruli detection and classification, interstitium and arteries detection | 111, human, PAS | CNN with U-net architecture | Highest Dice coefficient for the segmentation of healthy and segmentally sclerotic glomeruli, followed by the interstitium, capsule, and proximal tubuli classes; lower values for empty Bowman’s capsules, undefined tubuli, and atrophic tubuli; ICCs for glomerular counting by the CNN and the pathologists ranging 0.93-0.96; correlation coefficient for interstitium area 0.81 with R^2^=0.66 |
| Jayapandian et al., 2020 (USA) | Glomeruli, proximal and distal tubuli, arterioles, and capillaries detection | 459, human, H&E, PAS, trichomic, Silver | CNN segmentation-based model with U-net architecture | Performance measured with F-score ranging 0.81-0.95 (highest for glomeruli, lowest for capillaries) and best with PAS |
| Kato et al., 2015 (Japan) | Glomeruli detection | 20, rat, IHC | Segmental HOG | Precision of 0.874 in glomerular detection |
| Kawazoe et al., 2018 (Japan) | Glomeruli detection | 800, human, PAS, trichomic, Azan, silver | Faster R-CNN with a pretrained Inception-ResNet model | Precision of 0.93 with PAS e silver; long time to train the model; low computational time for test set |
| Lutnick et al., 2019 (USA) | Glomeruli detection | NS, mouse and human, PAS | Semantic segmentation CNN | Sensitivity 0.92, specificity 0.99, precision 0.93 and accuracy 0.99; H-AI-L method reduced the need for annotations |
| Maree et al., 2016 (France) | Glomeruli detection | 200 slides, human, trichromic | Cytomine and Icy softwares, combined color normalization  with ET-FL method | Average accuracy ranging 73-93% |
| Sarder et al., 2016 (USA) | Glomeruli detection | 15, rat, PAS | CNN with Gabor filtering textural analysis | Accuracy 0.86-0.87 for glomerular detection and 0.92 for segmentation of glomerular nuclei; higher speed than manual pathologists’ assessment |
| Simon et al., 2018 (USA) | Glomeruli detection | NS; mouse, rat, human; H&E, PAS, other | Supervised-learning CNN, SVM with LBP | High precision (>90%) in detection of glomeruli; robust to various stains |
| Temerinac-Ott et al., 2017 (France) | Glomeruli detection | NS, human nephrectomy and 20 biopsies; H&E, PAS, other | HOG and CNN patch-based | CNN better than HOG, H&E and PAS better than IHC |
| Zhao et al., 2016 (USA) | Glomeruli detection | NS, nonhuman primate, H&E | SVM | Average precision 78%, ranging 73-100% |

CNN, convolutional neural network; ET-FL, extremely randomized trees for feature Learning; FS, frozen section; H-AI-L, human AI loop; H&E, hematoxylin & eosin; HOG, histogram of oriented gradients; IHC, immunohistochemistry; LBP, local binary patterns; NS, not stated; PAS, Periodic-acid Schiff; SVM, support vector machine
